# Epidemiology and changed surgical treatment methods for fractures of the distal radius: A registry analysis of 42,583 patients in Stockholm County, Sweden, 2004–2010

**DOI:** 10.3109/17453674.2013.792035

**Published:** 2013-05-31

**Authors:** Maria K T Wilcke, Henrik Hammarberg, Per Y Adolphson

**Affiliations:** ^1^Division of Orthopaedics, Department of Clinical Sciences, Karolinska Institutet at Danderyd Hospital; ^2^Section of Hand Surgery, Department of Clinical Sciences and Education, Karolinska Institutet at Södersjukhuset, Stockholm, Sweden.

## Abstract

**Background and purpose:**

The incidence of fractures of the distal radius may have changed over the last decade, and operative treatment has been commoner during that time. We investigated the incidence of fractures of the distal radius and changing trends in surgical treatment during the period 2004–2010.

**Patients and methods:**

Registry data on 42,583 patients with a fracture of the distal radius from 2004 to 2010 were evaluated regarding diagnosis, age, sex, and surgical treatment.

**Results:**

The crude incidence rate was 31 per 10^4^ person-years with a bimodal distribution. After the age of 45 years, the incidence rate in women increased rapidly and leveled off first at a very high age. The incidence rate in postmenopausal women was lower than previously reported. In men, the incidence was low and it increased slowly until the age of 80 years, when it amounted to 31 per 10^4^ person-years. The number of surgical procedures increased by more than 40% despite the fact that there was reduced incidence during the study period. In patients ≥ 18 years of age, the proportion of fractures treated with plating increased from 16% to 70% while the use of external fixation decreased by about the same amount.

**Interpretation:**

The incidence rate of distal radius fractures in postmenopausal women appears to have decreased over the last few decades. There has been a shift in surgical treatment from external fixation to open reduction and plating.

The incidence of fractures of the distal radius was reported to increase from the 1950s until the 1990s, when it leveled off (Bengnér and Johnell 1985, Melton et al. 1998, Jónsson et al. 1999) and recent studies have suggested a declining incidence rate in postmenopausal women over the last 2 decades (Brogren et al. 2007, Lofthus et al. 2008, Sigurdardottir et al. 2011). Undisputedly, the incidence rate of fractures of the distal radius increases rapidly after menopause in women, but some authors have reported a continuous increase (Begnér and Johnell 1985, Mallmin and Ljunghall 1992, O’Neill et al. 2001, Thompson et al. 2004, Brogren et al. 2007, Flinkkilä et al. 2011) whereas others have described a leveling off of the incidence rate in older women (Alffram and Bauer 1962, Falch 1983, Miller and Evans 1985, Schmalholz 1988).

The first aim of this study was to determine the incidence of fractures of the distal radius in Stockholm County (with 2 million inhabitants) during the period 2004–2010. In the past decade, there has been a considerable increase in the use of volar locking plates for fixation of fractures of the distal radius (Koval et al. 2008, Chung et al. 2009, Mattila et al. 2011). Our second aim was to investigate the operative treatment used.

## Patients and methods

Data were extracted from the Stockholm County Council database for healthcare (the VAL database) for the period 2004–2010. The VAL database contains information on the healthcare of all individuals in Stockholm County. About 16 million outpatient visits and 310,000 inpatient visits are registered annually. The council pays healthcare providers according to their performance as reported in the database, thus ensuring the quality of the data. The VAL database is anonymous since the unique 10-digit national registration number of patients is encoded. Although it is not possible to identify anyone in the database, information on, for example, age, sex, and area of living is linked to each individual.

2004 was chosen as the first year of the study because from this time reporting was uniform and mandatory, and reimbursement was dependent on reporting of data by all healthcare providers in the county, thus guaranteeing a high coverage. Also, around 2004 the volar plate system was introduced for clinical use in Sweden.

The diagnoses were defined by the ICD 10 classification system (WHO 2007) where distal radius fractures (with or without a fractured ulnar styloid) are coded S52.50 (closed) or S52.51 (open) and distal radius and ulnar metaphyseal fractures are coded S52.60 (closed) or S52.61 (open). The first time an individual was reported with the diagnosis during the study period was noted, to avoid the risk of multiple counting. Non-residents who had received healthcare in Stockholm County for a fracture of the distal radius were excluded.

Population data were obtained from the database of Statistics Sweden (www.scb.se), a Swedish government agency. The population was defined as all individuals domiciled in the Stockholm County during 2004–2010.

The incidence rates were calculated as the number of reported first-time fractures divided by the total number of person-years of follow-up (population at risk) and expressed per 10^4^ person-years. Age- and sex-specific incidence rates were calculated. The data were stratified in 5 age classes (Tables 1 and 2). In addition, the incidence rate for women 50–79 years of age was calculated since changed epidemiology has been reported in this group in a recent Scandinavian report (Brogren et al. 2007).

**Table 1.** The number of individuals with a recorded fracture of the distal radius, the population at risk, the overall incidence rate per 10^4 ^person-years, and the average annual change in incidence rate (rate ratio) in Stockholm, Sweden, 2004–2010

Sex Age group	No. of fractures	Population at risk	Incidence rate (95% CI)	Rate ratio per (95% CI) ^a^	p-value year
Women					
≤ 17	6,071	1,449,812	42 (41–43)	0.95 (0.94–0.97)	< 0.01
18–39	1,938	2,113,882	9 (9–10)	0.99 (0.97–1.01)	0.3
40–64	7,779	2,210,100	35 (34–36)	0.98 (0.95–1.01)	0.2
65–79	5,636	730,046	77 (75–79)	0.96 (0.94–0.98)	< 0.01
≥ 80	4,321	392,148	110 (107–114)	0.97 (0.95–0.98)	< 0.01
Men					
≤ 17	9,844	1,525,859	64 (63–66)	0.95 (0.93–0.96)	< 0.01
18–39	2,175	2,121,935	10 (10–11)	0.98 (0.95–1.00)	0.06
40–64	3,044	2,223,532	14 (13–14)	0.98 (0.96–1.00)	0.06
65–79	1,158	620,522	19 (18–20)	0.94 (0.90–0.98)	< 0.01
≥ 80	617	200,683	31 (28–33)	0.96 (0.91–1.00)	0.06

**^a^** Analyzed with Poission regression.

**Table 2.** The annual number of first-recorded surgical interventions per 10^4^ inhabitants and the average annual increase (rate ratio) from 2004 through 2010 in Stockholm, Sweden

Sex Age group	2004	2005	2006	2007	2008	2009	2010	Rate ratio per year (95% CI)^a^	p-value
Women									
≤ 17	0.8	1.9	2.2	1.6	3.1	1.7	1.6	1.06 (0.94–1.19)	0.4
18–39	1.6	1.6	1.3	1.8	1.4	1.9	2.0	1.05 (1.02–1.08)	< 0.01
40–64	10.8	10.0	9.9	10.7	11.1	16.0	14.2	1.07 (1.03–1.12)	< 0.01
65–79	28.1	25.0	21.7	23.3	28.1	32.7	32.9	1.05 (1.01–1.09)	0.02
≥ 80	19.5	18.0	15.4	15.1	20.6	22.6	24.2	1.05 (1.01–1.10)	0.02
Men									
≤ 17	3.5	5.0	4.9	4.9	6.5	6.0	4.5	1.05 (0.98–1.12)	0.2
18–39	2.3	1.8	1.6	1.8	1.8	1.8	2.4	1.01 (0.94–1.08)	0.8
40–64	2.9	3.7	3.5	3.3	3.8	5.4	4.3	1.08 (1.03–1.13)	< 0.01
65–79	4.4	4.4	4.9	3.7	3.9	5.6	5.8	1.05 (1.01–1.08)	< 0.01
≥ 80	3.2	3.2	1.7	3.1	2.1	2.1	2.7	0.95 (0.90–1.01)	0.1
									
All patients	5.6	5.6	5.3	5.5	6.3	7.6	7.2	1.06 (1.03–1.08)	< 0.01

**^a^** Analyzed with Poission regression.

The surgical procedures were defined as they were classified by the Swedish version of the NOMESCO Classification of Surgical Procedures (NCSP), (NOMESCO 2008). We used the NCJ and NDJ group of codes to define fracture surgery. External fixation was searched as NCJ29 and NDJ29, percutaneous pinning as NCJ49 and NDJ49, and plating as NCJ69 and NDJ69. All surgical interventions of these types during the study period were taken into account for the analysis of changing surgical methods. When investigating changes in the quantity of surgical interventions, only the first surgical intervention was chosen to eliminate possible reoperations. Removal of fixation material was not considered as a surgical intervention.

The study was approved by the local Ethics Committee of Stockholm (DN 2012/2:6).

### Statistics

Incidence rates were calculated from mid-year population, which was estimated by taking the geometric mean of year-end population sizes of consecutive years. 95% confidence intervals (CIs) were calculated for the incidence rates using the Poisson exact method. Trends in incidence rates and operation rates were analyzed using Poisson regression. The assumption of the conditional mean being equal to the conditional variance was investigated by comparing the log-likelihoods of the negative binomial regression model with the Poisson regression model. Since there were some mild violations, robust standard errors were used as recommended by Cameron and Trivedi (2009). Chi-square test was used for comparison of proportions. Significance level was set at 0.05. Statistical analyses were performed with SAS software (SAS Institute Inc., Cary, NC, USA) and the R system, version 2.15.2 (R Foundation for Statistical Computing, Vienna, Austria).

## Results

During the study period of 7 years, 42,583 patients had a recorded diagnosis of a fracture of the distal radius, corresponding to an overall incidence rate of 31 per 10^4^ person-years (CI: 31–32). Mean age was 42 (0–106, SD 29) years for all patients during the study period, 51 (SD 28) years for women and 27 (SD 24) years for men. In adult patients (≥ 18 years of age), mean age was 64 (SD 17) years for women and 51 (SD 19) years for men.

In adults, the incidence rate was 25 per 10^4^ person-years (CI: 25–25). In adult women, the incidence rate was 36 per 10^4^ person-years (CI: 35–37) and in men it was 14 (CI: 13–14), resulting in a female-to-male incidence ratio of 2.7:1 (CI: 2.6–2.7). 38% of all reported fractures occurred in children (< 18 years), with an incidence rate of 53 per 10^4^ person-years (CI: 53–54).

**Figure 1.**
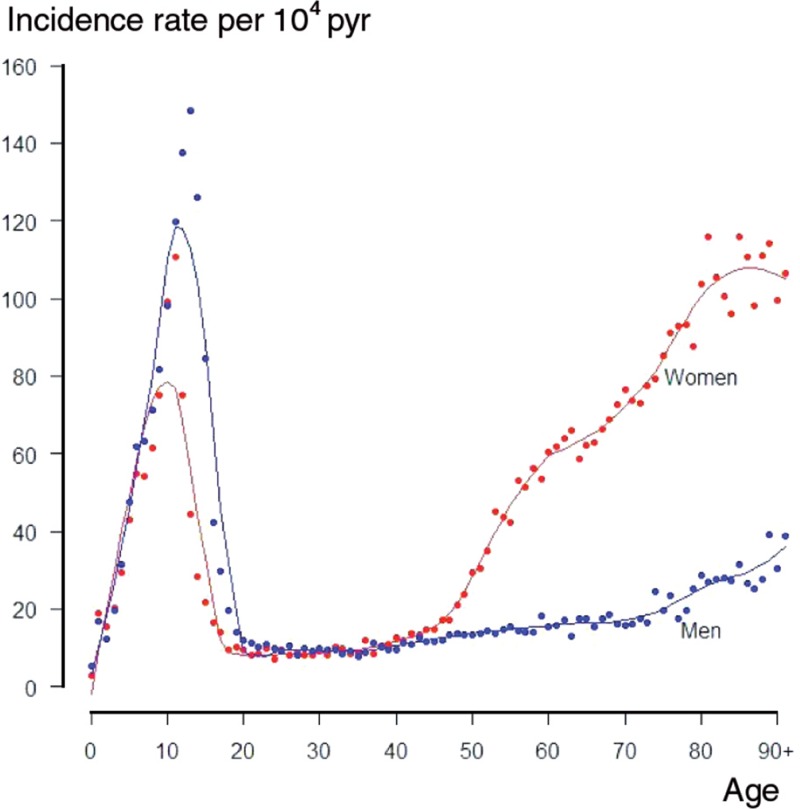
Incidence of reported fractures of the distal radius in Stockholm, Sweden, 2004–2010. Scatter plot with a loess curve inserted.pyr: person-years.

During the study period, the incidence rate decreased in children and in patients older than 65 years of age (Table 1). In women 50–79 years of age, the incidence rate was 60 per 10^4^ person-years (CI: 59–61). The age-related incidence rate of a reported fracture of the distal radius during the study period (Figure 1) was bimodal, with the first peak at the age of 11 in girls (incidence rate 111 per 10^4^ person-years, CI: 103–118) and 13 in boys (incidence rate 148 per 10^4^ person-years, CI: 140–157). After the childhood peak, the incidence rate increased with age, in women rapidly from menopause and in men slowly until high age. The second peak occurred after 80 years of age in both genders, but after this age the increase in incidence rate slowed down in women.

During the study period, 94,347 visits to health institutions associated with a recorded diagnosis of a fracture of the distal radius were reported, corresponding to a mean of 13,478 visits annually. A median of 2 (1–3) visits per patient were registered. 8% of the visits were inpatient care. The proportions of inpatient and outpatient care were similar in women and men (7.8% vs. 7.5%, p = 0.7). Patients aged 80 years and older were treated as inpatients to a greater extent than patients younger than 80 years (13% vs. 5–9% depending on age group, p < 0.01).

89% of all fractures were recorded as S52.50 or S52.51 and 11% were recorded as S52.60 or S52.61. S52.60 was recorded more in children (18%) than in adults (7%) (p < 0.01). 0.5% of the fractures were recorded as open fractures. There was no statistically significant difference in the proportion of open fractures between children and adults (0.2% vs. 0.8%, p = 0.4) or between women and men (0.7% vs. 1.0%, p = 0.5).

8,385 occasions of surgical interventions for distal radius fractures with the NCJ and NDJ codes investigated were reported. When only the first-recorded surgical intervention per patient was counted, the number was 7,541. The number of operations increased between 2004 and 2010 (Table 2).

**Figure 2.**
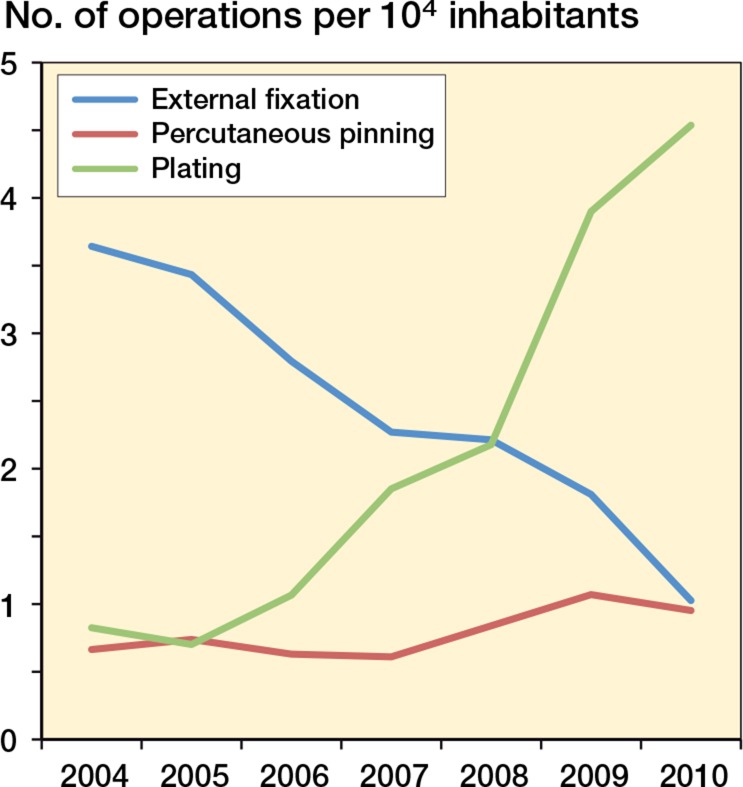
Changing surgical methods for fractures of the distal radius in adult patients (≥ 18 years old) in Stockholm, Sweden, 2004–2010.

In children (< 18 years), percutaneous pinning was the predominant method of fixation (used in 94% (89–97) of all surgical interventions). In adults with surgically fixated fractures, the proportion of fractures treated with open reduction and plating increased from 16% in 2004 to 70% in 2010—at the expense of the use of closed reduction and external fixation, which decreased from 71% to 16% during this period. The change occurred in all age groups and in both sexes (Figure 2).

External fixation was supplemented by percutaneous pins in 10% of the cases. Plating was reported with supplementary external fixation in 4% of the cases, with pins in 3%, and with both external fixation and pins in 0.4%.

A second or third surgical intervention was registered during the study period in 6% of the patients operated on with external fixation, in 6% of the plate fixated patients and in 3% of the patients operated on with percutaneous pinning.

## Discussion

In this paper we present population-based data from a large sample on the incidence rate of fractures of the distal radius and the numbers of different surgical interventions. Both inpatients and outpatients were included in this sample, which represented about one fifth of the Swedish population. We have only found 1 other study with a sample of similar size (Van Staa et al. 2001).

We found a lower incidence rate in women aged 50–79 years than in earlier investigations (Schmalholz 1988, Mallmin and Ljunghall 1992). Similar trends have recently been reported in Southern Sweden, Norway, and Iceland (Brogren et al. 2007, Diamantopoulus et al. 2011, Sigurdardottir et al. 2011). The prevention and treatment of osteoporosis could account for this change. Lofthus et al. (2008) found that while hormone replacement therapy increased, the incidence rate of fractures of the distal radius in middle-aged women decreased from 1979 to 1999 in a Norwegian population.

In the present study, the incidence rate in older women leveled off (Figure 1), but at a higher age than earlier reported (Alffram and Bauer 1962, Falch 1983, Miller 1985, Schmalholz 1988). Van Staa et al. (2001) reported a similar tendency, with leveling off in the very elderly, in a large sample of wrist fractures (46,947). The leveling off may have been delayed in the last 2–3 decades because of a more active lifestyle at higher age. Other recent reports have described a continuous rise in incidence rate even at a higher age (O’Neill et al. 2001, Thompson et al. 2004, Brogren et al. 2007, Flinkkilä et al. 2011). The leveling off is probably difficult to detect in small populations, which to some extent might explain the diverse findings.

In men, the incidence of fractures of the distal radius appears to be rather stable over time, and this finding is in accordance with both earlier and recent studies (Bengnér and Johnell 1985, Van Staa et al. 2001, Brogren et al. 2007, Lofthus et al. 2008). There was a decline in the incidence rate in men aged 65–79 years during the study period (Table 1). Whether or not this is an ongoing trend remains to be determined.

Hedström et al. (2010) reported a 13% increase in fractures in Swedish children from 1993 through 2007. Although their and our findings on the overall incidence rates of fractures of the distal radius in children are in accordance, we found an opposite trend with a declining incidence rate from 2004 through 2010 (Table 1). Perhaps this is a result of a less physically active lifestyle in children.

We found a shift in surgical methods from external fixation to plating in the last decade. The NOMESCO classification does not distinguish between dorsal and volar plating, but considering the development in the last decade, the increase in plating is presumably due to the use of volar plates. Other studies have shown similar trends in the USA and Finland (Koval et al. 2008, Chung et al. 2009, Mattila et al. 2011).

The number of fractures of the distal radius decreased from 2004 to 2010 while the number of surgical interventions increased (Tables 1 and 2). This indicates that the proportion of surgically treated fractures has increased. Similarly, more frequent operative fixation has been reported from the USA and Finland (Fanuele et al. 2009, Mattila et al. 2011).

We considered only the first-recorded fracture of the distal radius in an individual during the study period, to reduce the risk of multiple counting. The disadvantage is that patients who suffered more than 1 fracture of the distal radius during the period were only counted once, giving a risk of underestimation of the incidence rate. Another limitation of our data was the lack of information on the type of fracture (i.e. volar or dorsal displaced fracture, intra- or extra-articular fracture, and degree of displacement).

We included the S52.60 diagnosis since fractures of the distal radius with a fracture of the ulnar styloid may be coded as S52.50 or S52.60 in Sweden. S52.60 also covers the more unstable fractures of the distal radius and metaphyseal ulna. However, we decided that the advantage of not missing the fractures of the distal radius with an ulnar styloid fracture coded by S52.60 outweighed the disadvantage of including the less frequent distal radius and metaphyseal ulnar fractures.

The coding system does not distinguish between primary surgery and reoperations, so the reported surgical interventions represent both, which is a limitation. A comparison of all recorded occasions of surgical interventions with the number of first-recorded operations on a patient suggested that about 10% of the surgical interventions were reoperations related to the fracture or operations for a subsequent fracture of the distal radius during the period (data not shown).

The number and coding of diagnoses and surgical interventions in the VAL database should be accurate—given its direct correlation with reimbursement—and the coverage is presumably very high. For nonoperatively treated fractures, the coding and compensation systems were not uniform during the study period, and the registration was unreliable. Therefore, at this time, the VAL database could not be used to analyze conservative treatment for fractures of the distal radius.

In summary, our data support the suggestion that the incidence rate of fractures of the distal radius in postmenopausal women has decreased over the 2–3 last decades. A shift in surgical treatment from external fixation to open reduction and plating has occurred in this population.
